# Effects of antibiotic treatment on the fecundity of *Rhipicephalus haemaphysaloides* ticks

**DOI:** 10.1186/s13071-018-2807-7

**Published:** 2018-04-13

**Authors:** Lan-Hua Li, Yi Zhang, Dan Zhu

**Affiliations:** 1 School of Publish Health and Management, Weifang Medical University, Weifang, 261053 People’s Republic of China; 2 National Institute of Parasitic Diseases, Chinese Center for Disease Control and Prevention, WHO Collaborating Centre for Malaria, Schistosomiasis and Filariasis, Key Laboratory of Parasite & Vector Biology, Ministry of Health, Shanghai, 200025 People’s Republic of China

**Keywords:** *Rhipicephalus haemaphysaloides*, Endosymbiont, *Coxiella*, *Rickettsia*, Fecundity

## Abstract

**Background:**

Endosymbiotic bacteria inhabit a variety of arthropods including ticks and may have multiple effects on the host’s survival, reproduction or pathogen acquisition and transmission. *Rhipicephalus haemaphysaloides* is one of the most widely distributed tick species in China. The symbiotic bacteria composition and their impacts to *R. haemaphysaloides* ticks have not been studied. The present study investigated the composition of microbial community in *R. haemaphysaloides* ticks and then assessed the effects of endosymbionts on the host’s fecundity by antibiotic treatment experiments.

**Methods:**

The microbial population of female and male *R. haemaphysaloides* ticks was analyzed using Illumina Miseq sequencing of *16S* rRNA gene. Thirty engorged female ticks were then randomly divided into five groups and injected with ampicillin, ciprofloxacin, kanamycin, tetracycline, or phosphate-buffered solution (PBS), respectively. Effects of antibiotic treatments on maternal oviposition, egg hatching and density of endosymbionts were evaluated.

**Results:**

Illumina Miseq sequencing showed that *Coxiella* and *Rickettsia* were the predominant bacterial genera inhabiting *R. haemaphysaloides* ticks. Antibiotic treatment experiments found that kanamycin reduced the density of *Coxiella*-like endosymbiont (*Coxiella*-LE hereafter) in eggs, ciprofloxacin reduced the density of *Rickettsia*-like endosymbiont (*Rickettsia*-LE), and tetracycline had effect on both endosymbionts, while ampicillin affected neither. Meanwhile hatching rates of eggs were observed to decrease greatly in the kanamycin or tetracycline-treated group but maintained in the ampicillin or ciprofloxacin-treated group. Furthermore, the reduced hatching rates were found to be associated with density of *Coxiella*-LE in eggs.

**Conclusions:**

The findings indicate that *Coxiella*-LE is essential for the reproduction of *R. haemaphysaloides* ticks, and that kanamycin can be used to study the role of *Coxiella*-LE on ticks.

## Background

Endosymbiotic bacteria inhabit a variety of arthropods. Some bacteria exhibit multiple effects on the host’s survival, reproduction or pathogen acquisition and transmission [1]. Ticks are obligate hematophagous arthropods and are considered second only to mosquitoes as vectors of human disease in the world [2]. To date, endosymbiotic bacteria of the genera *Coxiella*, *Rickettsia*, *Francisella*, *Wolbachia*, *Arsenophonus* or “*Candidatus* Midichloria mitochondrii” have been found in ticks [3]. Some of them were found to have important roles in ticks’ survival or reproduction. Zhong et al. [4] suggested that reproductive fitness of *A. americanum* was reduced after treated with rifampin or tetracycline, and reduction in fecundity was probably related to the density of *Coxiella*-like endosymbiont (*Coxiella*-LE). However, they could not rule out the contribution of the *Rickettsia* to the effects because the antibiotics used in the study (rifampin and tetracycline) could reduce the density of *Coxiella*-LE and *Rickettsia*-like endosymbiont (*Rickettsia*-LE) simultaneously. In other studies, Zhang et al. [5] found that reproductive fitness of *H. longicornis* decreased dramatically after the density of *Coxiella*-LE was reduced by tetracycline treatment, while Andre et al. [6] revealed that although the density of *Rickettsia*-LE decreased significantly after treatment with tetracycline or ciprofloxacin, it did not affect the fecundity of *I. pacificus* ticks. Meanwhile “*Candidatus* Midichloria mitochondrii” was found to have the ability to impact the process of engorgement and molt in *I. ricinus* ticks [7]. In addition, bacterial symbionts can affect pathogen acquisition and transmission of ticks. For example, disturbing microbiota by antibiotic treatment could affect the susceptibility of *I. scapularis* ticks to *Borrelia burgdorferi*, *Anaplasma phagocytophilum*, *A. marginale* and *Francisella novicida* [8–10]. However, the role of tick endosymbionts remains poorly understood because the existing studies are limited to a few tick species [11].

*Rhipicephalus haemaphysaloides* is widely distributed in China [12, 13]. It has been reported to be vector of several pathogens including Kyasanur Forest disease virus, *Babesia microti*, *Ehrlichia canis* and so on [14, 15]. In tick species of *Rhipicephalus* genus, bacterial communities in *R. turanicus*, *R. sanguineus*, *R. annulatus* and *R. microplus* have been analyzed [11, 16–18]. *Coxiella*, *Rickettsia*, *Wolbachia* and “*Candidatus* Midichloria mitochondrii” have been reported in *Rhipicephalus* ticks. However, the symbiotic bacterial composition of *R. haemaphysaloides* and their impacts to the hosts have not been studied. In order to test whether endosymbiont influence the fecundity of *R. haemaphysaloides*, we assessed the effects of endosymbionts on maternal oviposition and hatching of eggs by antibiotic treatment experiments.

## Methods

### Ticks

An engorged *R. haemaphysaloides* female tick was removed from a dog in Tengchong County, Yunnan Province of China. The colony was then maintained in the laboratory in an incubator at 25 °C, with 85% relative humidity and a 14/10 h light/dark photoperiod regimen as described previously [14]. Adult ticks were fed on New Zealand white rabbit.

### Microbial population analysis

The DNA of unfed female or male ticks was extracted using DNeasy Blood & Tissue Kit (Qiagen, Hilden, Germany) according to the manufacturer’s protocol.

DNA samples of 15 female or 15 male ticks were pooled together in equal concentrations. The microbial population of females and males were then analyzed separately using Illumina MiSeq sequencing by Shanghai Majorbio Bio-pharm Technology Company Limited (Shanghai, China). In brief, the V3-V4 variable region of the bacterial *16S* rRNA gene was amplified by PCR using the universal primers 338F and 806R (Table 1) under conditions of 95 °C for 2 min, followed by 25 cycles at 95 °C for 30 s, 55 °C for 30 s and 72 °C 30 s with a final extension at 72 °C for 5 min [19]. PCR amplifications were performed in quintuplicate for each sample. Purified PCR products were pooled together in equal molar concentrations and sequenced by Illumina MiSeq platform according to standard protocols. Sequences with 97% similarity were clustered into the same operational taxonomic units (OTUs), with one sequence per OTU being selected as a representative sequence for further downstream analysis [8]. For each representative sequence, the GreenGene Database was applied in annotating taxonomic information with RDP classifier (v.2.2). Alpha diversity was estimated using the ACE, Chao1, Shannon’s and Simpson’s indices.

**Table 1:** Primers for PCR amplification and qPCR reaction

Organism	Target gene	Primer	Sequence (5'-3')	Reference
Bacteria	*16S*	338f	ACTCCTRCGGGAGGCAGCAG	[19]
		806r	GGACTACCVGGGTATCTAAT	
*R. haemaphysaloides*	Actin gene	Ractin-F	GTGCCCATCTACGAAGGTTAC	This study
		Ractin-R	CCATCTCCTGCTCGAAGTCC	
*Rickettsia*-like endosymbiont	Citrate synthase gene (*gltA*)	gltA-F	TCCTACATGCCGACCATGAG	[20]
		gltA-R	AAAGGGTTAGCTCCGGATGAG	
*Coxiella*-like endosymbiont	*16S* rRNA gene	L-CoxF	TGAGTGTTGACGTTACCCACAG	This study
		L-CoxR	GCATTTCACCGCTACACCG	

### Antibiotic treatments

In total, 30 engorged female ticks dropping from the rabbits were weighed and then randomly divided into five treatment groups: ampicillin, ciprofloxacin, kanamycin, tetracycline, and phosphate buffered solution (PBS), with six ticks in each group. Antibiotic solutions were made at concentrations of 10 mg/ml. Each engorged tick was injected into the hemocoel between the first and second legs using microinjection needles. The doses of each solution used for injection were 1μl per 100 mg body weight of ticks. The needle was left inside tick body for 30 s after injection and was then withdrawn slowly [4].

Each injected tick was maintained in separate containers and monitored for oviposition daily. The period between dropping from the rabbit and the beginning of oviposition was recorded as time to oviposition. Ticks dying before oviposition were excluded from the study. Egg masses of each tick were weighed after completion of oviposition. The ratio of two weights, the total weight of egg mass to the weight of the engorged female before oviposition, was recorded as the oviposition index. After that, 100 randomly selected eggs from each female were preserved in 75% ethanol for further DNA extraction and molecular analysis. Another 200 randomly selected eggs from each female were put into a new container to estimate hatching rate. The remaining eggs were left in the original containers. Hatching states of eggs were monitored daily. The incubation period for eggs was defined as time from deposition of the first egg to the occurrence of the first larvae. For each container with 200 eggs, the number of hatched larvae was counted under a dissecting microscope after being frozen, and the percentage of hatched larvae was defined as the hatching rate for eggs.

### Quantitative polymerase chain reaction (qPCR) assay

DNA of eggs from each group was extracted using the method described above. Relative densities of *Coxiella*-LE and *Rickettsia*-LE in eggs were analyzed using the SYBR green qPCR approach. The *16S* rRNA gene fragment of *Coxiella*-LE, citrate synthase (*gltA*) gene fragment of *Rickettsia*-LE and the beta-actin biosynthetic (actin) gene fragment of *R. haemaphysaloides* ticks were qPCR-amplified (Table 1). The relative density of *Coxiella*-LE or *Rickettsia*-LE was defined as *Coxiella 16S* rRNA or *Rickettsia gltA* gene copies per tick actin gene copy [20]. Specificity of all the primers was verified by PCR amplification and then sequencing of the PCR products. To further confirm the prevalence of *Coxiella*-LE and *Rickettsia*-LE in adult ticks, DNA samples of adult ticks were also analyzed separately by qPCR.

All qPCR reactions were performed in CFX96 Real-Time PCR System (Bio-Rad laboratories Incorporation, Richmond, CA, USA). Each 15 μl reaction mixture contained 1.5 μl DNA sample, 7.5 μl qPCR Master Mix Plus (Takara Bio Inc, Shiga, Japan), 1 μl of 5 mM primers and 5 μl of water. Four negative controls were used in each 96-well qPCR plate to exclude contamination. The cycling condition for each reaction was 95 °C for 30 s, 40 cycles of 95 °C for 5 s and 60 °C for 30 s, with the fluorescence recorded at the annealing stage.

### Statistical analysis

Statistical analysis was carried out with SPSS 19.0 software (IBM, Armonk, NY, USA). The relative density of *Coxiella*-LE and *Rickettsia*-LE was log-transformed for significance testing. One-way ANOVA was used to compare weight of ticks, oviposition index, log-transformed relative density of *Coxiella*-LE or *Rickettsia*-LE and hatching rate among groups. Difference between any of the antibiotic-treated group and PBS-treated group was analyzed by Dunnett’s test. Time to oviposition and incubation period of eggs among groups were compared with the Kruskal-Wallis H-test, and Dunn’s test was used to analyze the difference between antibiotic-treated and PBS-treated groups. Spearman’s rank correlation and multiple linear regression model were used to analyze the association between relative density of *Coxiella*-LE or *Rickettsia*-LE and incubation period or hatching rate for eggs. Significant difference was defined as *P* < 0.05 with a two-tailed test.

## Results

### Microbial community composition in unfed *R. haemaphysaloides* adult ticks

**Fig. 1:**
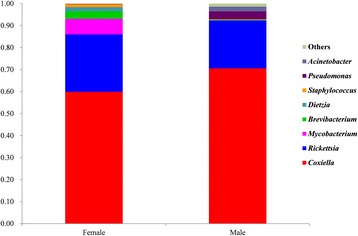
The relative abundance of bacterial genera in unfed female and male *Rhipicephalus haemaphysaloides* ticks

In total, 38,599 sequence reads were obtained from females and 41,109 reads from males of *R. haemaphysaloides* after trimming and removing all low-quality sequences. Twenty-four different bacterial genera were identified from female ticks and 57 genera were identified from males. Among all the bacterial genera, 21 were shared by both females and males. Bacterial genera with relative abundance higher than 1% in female or male ticks including *Coxiella*, *Rickettsia*, *Mycobacterium*, *Brevibacterium*, *Pseudomona*s, *Acinetobacter*, *Dietzia* and *Staphylococcus*. *Coxiella* and *Rickettsia* were the predominant genera (Fig. 1). The results of qPCR further revealed that prevalences of both *Coxiella*-LE and *Rickettsia*-LE were 100% in adult ticks. Except for *Coxiella* and *Rickettsia*, all the above genera were extracellular bacteria and were probably derived from the guts of ticks or the environment.

Alpha diversity of female and male ticks is shown in Table 2. Community richness revealed by ACE and Chao1 estimators was higher in male ticks than that in females; however, community diversity estimated by Shannon’s and Simpson’s indices was higher in females than that in males.

**Table 2:** Alpha-diversity (confidence interval) of female and male *R. haemaphysaloides* ticks

Ticks	ACE estimator	Chao1 estimator	Simpson’s index	Shannon’s index	Coverage (%)
Female	37.47 (33.45–52.67)	35.50 (32.65–50.89)	0.43 (0.43–0.44)	1.13 (1.12–1.14)	99.98
Male	75.08 (71.58–86.40)	72.77 (70.56–83.60)	0.55 (0.54–0.55)	0.93 (0.92–0.94)	99.98

### Effects of antibiotic treatment on tick fecundity and density of *Coxiella*-LE and *Rickettsia*-LE

One tick from the tetracycline group died without laying eggs and was excluded from data analysis. The average weight of engorged ticks among five groups was not statistically different (data not shown).

**Fig. 2:**
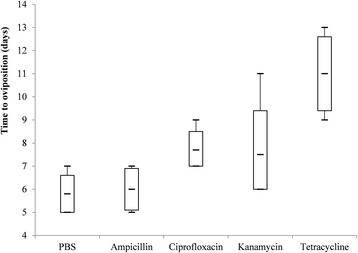
Box-and-whisker plots of the time to oviposition in five treatment groups of *R. haemaphysaloides*

**Fig. 3:**
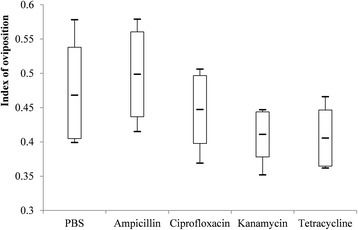
Box-and-whisker plots of the index of oviposition in five treatment groups of *R. haemaphysaloides*

**Fig. 4:**
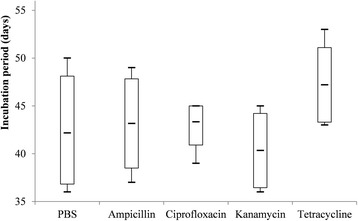
Box-and-whisker plots of the incubation period of eggs in five treatment groups of *R. haemaphysaloides*

**Fig. 5:**
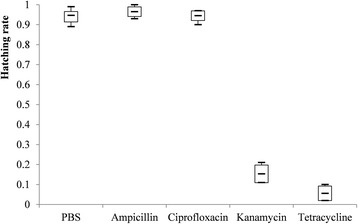
Box-and-whisker plots of the hatching rate of eggs in five treatment groups of *R. haemaphysaloides*

**Fig. 6:**
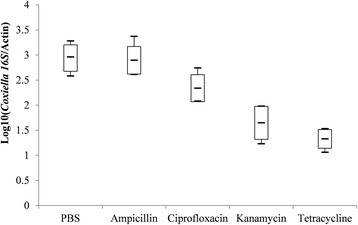
Box-and-whisker plots of log-transformed relative density of *Coxiella*-like endosymbiont in five treatment groups of *R. haemaphysaloides*

**Fig. 7:**
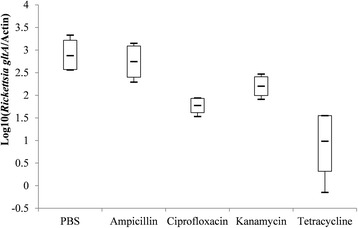
Box-and-whisker plots of log-transformed relative density of *Rickettsia*-like endosymbiont in five treatment groups of *R. haemaphysaloides*

Indicators of the fecundity and relative density of *Coxiella*-LE and *Rickettsia*-LE in eggs from each group are summarized in Table 3 and Figs. 2, 3, 4, 5, 6 and 7.

When compared with PBS-treated group, time to oviposition was delayed in tetracycline-treated group (Dunn’s test: *Z* = 18.98, *P* = 0.002), but it was not significantly changed in ampicillin-, ciprofloxacin- and kanamycin-treated groups (Fig. 2). Oviposition index and incubation period of eggs were not statistically different among five treatment groups (Figs. 3 and 4). However, hatching rates of eggs in the kanamycin-treated group (15.3%) and tetracycline-treated group (5.6%) were significantly lower than that in the PBS- (94.7%), ampicillin- (96.5%) or ciprofloxacin-treated (94.5%) groups (Table 3 and Fig. 5).

**Table 3:** Effects of antibiotic treatment on fecundity *R. haemaphysaloides* and density of *Coxiella*-LE and *Rickettsia*-LE in eggs

Treatment	No. of Ticks	Time to oviposition (days)	Index of oviposition	Incubation period (days)	Hatching rate	Log-transformed relative density of *Coxiella*-LE	Log-transformed relative density of *Rickettsia*-LE
PBS	6	5.8 ± 0.8	0.5 ± 0.06	42.2 ± 5.4	0.95 ± 0.03	3.0 ± 0.3	2.9 ± 0.3
Ampicillin	6	6.0 ± 0.9	0.5 ± 0.05	43.2 ± 4.7	0.97 ± 0.02	2.9 ± 0.3	2.8 ± 0.4
Ciprofloxacin	6	7.7 ± 0.8	0.4 ± 0.03	43.3 ± 2.4	0.95 ± 0.02	2.3 ± 0.3	1.8 ± 0.2^a^
Kanamycin	6	7.5 ± 1.9	0.5 ± 0.06	40.3 ± 3.9	0.15 ± 0.04^a^	1.7 ± 0.3^a^	2.2 ± 0.2
Tetracycline	5	11.0 ± 1.6^a^	0.4 ± 0.04	47.2 ± 3.9	0.06 ± 0.04^a^	1.3 ± 0.2^a^	1.0 ± 0.7^a^
*F*-value		–	3.30	–	307.8	39.4	24.6
*P*-value		< 0.01^b^	0.03	0.16^c^	< 0.01	< 0.01	< 0.01

^a^The value is statistically different from that of PBS-treated group

^b^Kruskal-Wallis H-test: *χ*^*2*^ = 16.459, *df* = 4

^c^Kruskal-Wallis H-test: *χ*^*2*^ = 6.534, *df* = 4

As shown in Fig. 6, the estimated relative density of *Coxiella*-LE in eggs of both the tetracycline- and kanamycin-treated ticks were lower than that of PBS-treated ticks, while it was not statistically different between ampicillin- or ciprofloxacin-treated groups and PBS-treated groups. Meanwhile, the relative density of *Rickettsia*-LE in eggs was reduced by ciprofloxacin or tetracycline treatment, but not affected by kanamycin or ampicillin injection when comparing to PBS treatment (Fig. 7).

### Association between density of *Coxiella*-LE or *Rickettsia*-LE and egg hatching

As shown in Table 4, the hatching rate of eggs was positively correlated with the relative density of both *Coxiella*-LE and *Rickettsia*-LE in eggs (Spearman’s correlation coefficient = 0.89, *P* < 0.001 for *Coxiella*-LE; Spearman’s correlation coefficient = 0.57, *P* < 0.001 for *Rickettsia*-LE). Multiple linear regression analysis was conducted to analyze the separate effects of *Coxiella*-LE and *Rickettsia*-LE. It revealed that only the density of *Coxiella*-LE was associated with hatching rate, but there was no association for *Rickettsia*-LE (Table 5).

**Table 4:** Spearman’s correlation coefficient between relative density of *Coxiella*-LE or *Rickettsia*-LE and egg hatching of *R. haemaphysaloides*

Variables	Incubation period	Hatching rate	Log(Cox16S/Actin)
Log(Ric-gltA/Actin)	-0.27	0.57^*^	0.76*
Log(Cox16S/Actin)	-0.20	0.89^*^	–

^*^P < 0.001

**Table 5:** The influence of *Coxiella*-LE and *Rickettsia*-LE on hatching rate of *R. haemaphysaloides* in multiple linear regression model

Variables	Coefficient	Standard error	Standard coefficient	*t*-value	*P-*value
Intercept	-0.53	0.12		-4.4	< 0.001
Log (Ric-gltA/Actin)	-0.14	0.07	-0.26	-2.0	0.06
Log (Cox16S/Actin)	0.65	0.08	1.08	8.3	< 0.001

## Discussion

The microbiome of *R. haemaphysaloides* ticks has not been studied previously. In the present study, microbial communities of adult *R. haemaphysaloides* ticks were analyzed based on the Illumina Miseq sequencing of *16S* rRNA gene. Consistent with the results in *R. sanguineus* and *R. turanicus* ticks [16, 17], our study revealed that *Coxiella* and *Rickettsia* were the predominant genera of both female and male *R. haemaphysaloides* ticks. However, in *R. annulatus* and *R. microplus* ticks, neither *Coxiella* nor *Rickettsia* was the predominant symbiotic bacteria [18, 21]. Interestingly, within *Rhipicephalus*, both *R. annulatus* and *R. microplus* belong to the subgenus of *Boophilus*, while *R. sanguineus*, *R. turanicus* and *R. haemaphysaloides* belong to another phylogenic clade [22]. Therefore, the differences of microbiome among tick species may reflect the co-evolution of symbionts with host. It can be argued that wild collected *R. haemaphysaloides* ticks may have a different microbiome composition with laboratory reared ticks. However, it is rational to speculate that *Coxiella* and *Rickettsia* are also the predominant genera for wild collected ticks because both *Coxiella*-LE and *Rickettsia*-LE are transovarially transmitted while extracellular bacteria are often obtained from the environment [23]. The speculation was also confirmed by microbial community analysis of 13 engorged female *R. haemaphysaloides* ticks removed from domestic animals in Tengchong County. The results showed that *Coxiella*-LE, *Rickettsia*-LE and pathogenic *Anaplasma* were the most predominant bacteria in the field collected samples, with relative abundances of 68.5, 12.6 and 26.4%, respectively (data not shown).

The difference of microbial diversity between female and male ticks was studied previously in *Dermacentor occidentalis*, *I. scapularis* and *A. americanum* ticks [24]. The results showed that female ticks harbored a less diverse array of bacteria than males. The authors supposed that higher abundance of *Rickettsia*-LE and *Francisella* endosymbionts led to lower community diversity in female ticks than males because endosymbionts were thought to partially exclude the invasion of other bacteria. Unlike the previous study, we found that community richness was higher in male ticks than females, while community diversity was higher in females than males (Table 2). However, the biological significance of these differences still needs to be investigated.

To date, a limited number of studies have been conducted to investigate the effects of endosymbionts on fecundity of ticks. In the present study, engorged female *R. haemaphysaloides* ticks were treated with one of the five solutions including ampicillin, ciprofloxacin, kanamycin, tetracycline and PBS. The results showed that kanamycin reduced the density of *Coxiella*-LE in eggs and ciprofloxacin reduced the density of *Rickettsia*-LE, while tetracycline had effect on both endosymbionts and ampicillin had effect on neither (Table 2). Moreover, the hatching rates of eggs were observed to decrease greatly in the kanamycin- or tetracycline-treated groups but it maintained in the ciprofloxacin- or ampicillin-treated groups. Therefore, we suggested that *Coxiella*-LE was related to the reduced hatching rates of eggs, and that it was a primary endosymbiont of *R. haemaphysaloides*. Meanwhile, *Rickettsia*-LE was probably a secondary endosymbiont of ticks [6]. Consistent with the previous study, time to oviposition was delayed in the tetracycline-treated group [4, 6]. It was supposed that delaying oviposition in ticks by tetracycline treatment should lie in reasons other than the clearance of *Coxiella*-LE, because delaying oviposition was not observed in ticks treated with kanamycin. Thus we suspected tetracycline might influence ticks by certain unknown mechanism, and kanamycin was considered to be the better choice to disturb *Coxiella*-LE than tetracycline in related studies. In addition, since ampicillin does not have an effect on either of the two endosymbionts, it can be used to study the function of gut microbiome in *R. haemaphysaloides* ticks. Distinct from the study on *A. americanum* ticks [4], the index of oviposition and the incubation period of larvae were not significantly altered by any of the antibiotics. The reason might lie in the relatively limited number of ticks used in our study. The present study can be challenged that it cannot exclude completely the impact of symbiotic bacteria other than *Coxiella*-LE and *Rickettsia*-LE on the reproduction. However, considering their vertical transmitted feature, endosymbionts are more probable to manipulate reproduction of ticks than extracellular bacterial symbionts.

## Conclusions

Our study found that *R. haemaphysaloides* ticks harbored two endosymbionts, namely *Coxiella*-like and *Rickettsia*-like endosymbionts; the *Coxiella*-like endosymbiont was considered to be related to fecundity of *R. haemaphysaloides* ticks and kanamycin was considered to be suitable antibiotic to manipulate the density of *Coxiella*-like endosymbiont.

## Abbreviations

*Coxiella*-LE: *Coxiella*-like endosymbiont
gltA: citrate synthase gene
OTU: operational taxonomic unit
PBS: phosphate buffer saline
RDP: Ribosomal Database Project
*Rickettsia*-LE: *Rickettsia*-like endosymbiont
